# Cardiovascular variability, sociodemographics, and biomarkers of disease: the MIDUS study

**DOI:** 10.3389/fphys.2023.1234427

**Published:** 2023-08-24

**Authors:** Tara Gruenewald, Teresa E. Seeman, Tse-Hwei Choo, Jennifer Scodes, Clayton Snyder, Martina Pavlicova, Maxine Weinstein, Joseph E. Schwartz, Ramakrishna Mukkamala, Richard P. Sloan

**Affiliations:** ^1^ Department of Psychology, Chapman University, Orange, CA, United States; ^2^ David Geffen School of Medicine, University of California Los Angeles, Los Angeles, CA, United States; ^3^ Mental Health Data Science Division, New York State Psychiatric Institute, New York, NY, United States; ^4^ Department of Biostatistics, Mailman School of Public Health, Columbia University Irving Medical Center, New York, NY, United States; ^5^ Georgetown University, Washington, DC, United States; ^6^ Renaissance School of Medicine, Stony Brook University, New York, NY, United States; ^7^ Department of Medicine, Columbia University Irving Medical Center, New York, NY, United States; ^8^ Swanson School of Engineering, University of Pittsburgh, Pittsburgh, PA, United States; ^9^ Division of Behavioral Medicine, Department of Psychiatry, Columbia University Irving Medical Center, New York, NY, United States; ^10^ New York State Psychiatric Institute, New York, NY, United States

**Keywords:** blood pressure variability, sociodemographic, biomarker, mortality, cardiovascular dynamics

## Abstract

**Introduction:** Like heart rate, blood pressure (BP) is not steady but varies over intervals as long as months to as short as consecutive cardiac cycles. This blood pressure variability (BPV) consists of regularly occurring oscillations as well as less well-organized changes and typically is computed as the standard deviation of multiple clinic visit-to-visit (VVV-BP) measures or from 24-h ambulatory BP recordings (ABPV). BP also varies on a beat-to-beat basis, quantified by methods that parse variation into discrete bins, e.g., low frequency (0.04–0.15 Hz, LF). However, beat-to-beat BPV requires continuous recordings that are not easily acquired. As a result, we know little about the relationship between LF-BPV and basic sociodemographic characteristics such as age, sex, and race and clinical conditions.

**Methods:** We computed LF-BPV during an 11-min resting period in 2,118 participants in the Midlife in the US (MIDUS) study.

**Results:** LF-BPV was negatively associated with age, greater in men than women, and unrelated to race or socioeconomic status. It was greater in participants with hypertension but unrelated to hyperlipidemia, hypertriglyceridemia, diabetes, elevated CRP, or obesity. LF-diastolic BPV (DBPV), but not-systolic BPV (SBPV), was negatively correlated with IL-6 and s-ICAM and positively correlated with urinary epinephrine and cortisol. Finally, LF-DBPV was negatively associated with mortality, an effect was rendered nonsignificant by adjustment by age but not other sociodemographic characteristics.

**Discussion:** These findings, the first from a large, national sample, suggest that LF-BPV differs significantly from VVV-BP and ABPV. Confirming its relationship to sociodemographic risk factors and clinical outcomes requires further study with large and representative samples.

## Introduction

For decades, it has been widely recognized that lower heart rate (HR) is associated with reduced morbidity and mortality (Benetos et al., 1999; Seccareccia et al., 2001; Diaz et al., 2005; Tverdal et al., 2008; Cooney et al., 2010). But HR is not stable—it fluctuates around the mean. Beginning in the 1970’s, research began to reveal that these fluctuations were not random noise but were organized into distinct periodicities, typically quantified by Fourier-based spectral analysis, that reflect underlying autonomic physiology and have prognostic significance. Oscillations in the high frequency range (0.15–0.40 Hz, HF) are widely accepted to reflect the activity of the parasympathetic nervous system (PNS). Low frequency oscillations (0.04–0.15 Hz, LF) are the product of both the PNS and the sympathetic nervous system (SNS). Very low frequency oscillations (0.003–0.03 Hz, VLF) are less well understood but are thought to reflect thermoregulatory factors and the renin-angiotensin system.

This “heart rate variability” (HRV) predicts outcomes following myocardial infarction (Kleiger et al., 1987; Bigger et al., 1992) or diagnosis of heart failure (La Rovere et al., 2003), progression of atherosclerosis in patients with coronary artery disease (CAD) (Huikuri et al., 1999), and the development of CAD in healthy community samples (Tsuji et al., 1996; Liao et al., 1997). Community studies have explored its relationship to sociodemographic and psychosocial factors to understand the contextual factors that drive risk of CAD (Liao et al., 1997; Hemingway et al., 2005; Sloan et al., 2008; O'Neal et al., 2016). HRV has become a valuable index to test the role of the autonomic nervous system in inflammation (Sloan et al., 2007; Jarczok et al., 2014; Samniang et al., 2016), the cardioprotective effects of exercise training (Hamer and Steptoe, 2007; Souza et al., 2007; Masson et al., 2015; Dor-Haim et al., 2017), gastrointestinal disorders (Waring et al., 2004; Cheng et al., 2013; Jang et al., 2017), and cognitive function and neurodegenerative disorders (Kim et al., 2006; Nicolini et al., 2014; LeBouthillier & Asmundson, 2017). Thus, measurement “noise” in HR is now recognized as a valuable indicator of health.

**FIGURE 1:**
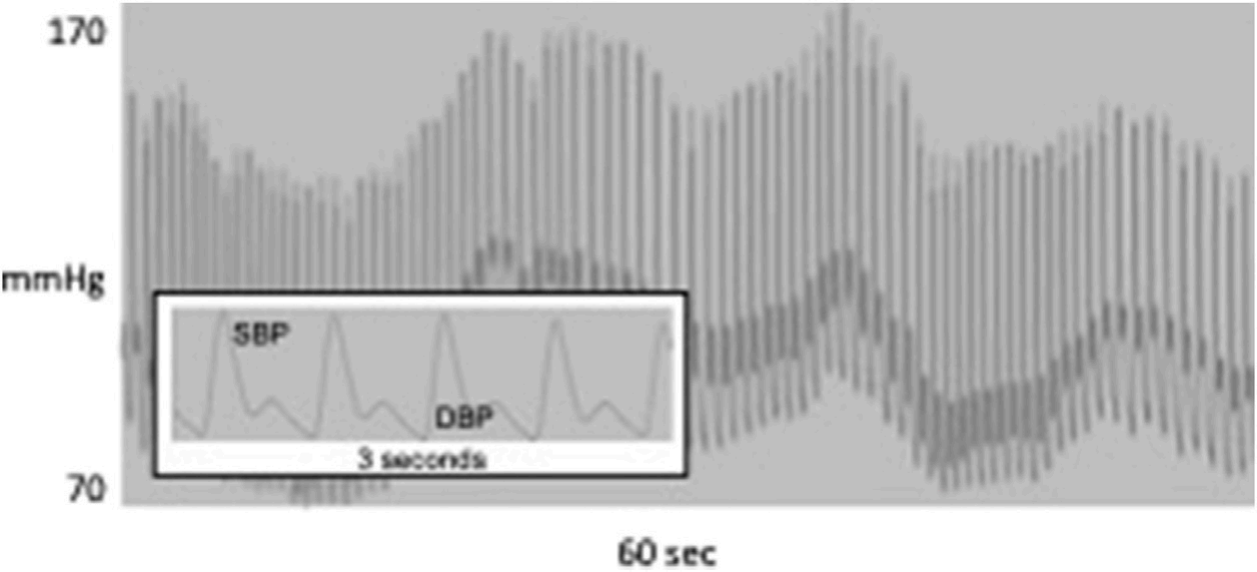
60 s of continuous blood pressure waveform showing oscillation at about 0.05 Hz; inset shows systolic and diastolic BP for 3 s.

Today, blood pressure (BP) is in the same position as HR was 40 years ago. While the clinical significance of an individual’s BP level has long been accepted (Lewington et al., 2002), within-subject BP variability (BPV), once also dismissed as noise, is now thought to contain valuable information (Schillaci et al., 2011). However, the time scale of BPV, as typically assessed, is considerably greater than that for HRV. Measured repeatedly over weeks or even years, the standard deviation (SD) of visit-to-visit (VVV) BP was associated with vascular function (Diaz et al., 2011), predicted the development of cardiovascular events in hemodialysis (Rossignol et al., 2012), stroke (Shimbo et al., 2012), hypertension (Chen et al., 2011), cognitive decline (Guo et al., 2016) and the risk of dementia (Rouch et al., 2020), and mortality (Muntner et al., 2011; Muntner et al., 2015b). In a study of 259 patients referred to a hypertension clinic, beat-to-beat BP was measured along with ambulatory and visit-to-visit BP (Wei et al., 2014). The authors reported direct relationships between target organ damage and beat-to-beat BPV but importantly, BPV was measured using time domain statistics reflecting global variability instead of frequency-specific LF-BPV. Similarly, in 92 patients with CT-confirmed acute ischemic strokes, beat-to-beat BP also was measured but BPV was computed only in the time domain as the standard deviation (Dawson et al., 2000). On a 24-h scale, the SD of BP measured every 30 min by ambulatory (ABPV) monitoring was associated with cardiovascular mortality after 8.5 years follow-up (Kikuya et al., 2000) and with greater target organ damage (Palatini et al., 1992). On a still shorter time scale, BP varies on a beat-to-beat basis, in the same frequency range as HRV. Figure 1 shows a BP oscillation at about 0.05 Hz, corresponding to the LF band of HRV. BP also oscillates in the high frequency range (0.15–0.40 Hz), but these oscillations are driven primarily by intrathoracic pressure changes associated with respiration and therefore have been of little interest. LF-BPV, however, is only poorly understood and may have greater clinical and physiological significance.

Some have suggested that the ultimate prognostic value of BPV may require analysis of these beat-to-beat BP oscillations (Hansen et al., 2009). However, because noninvasive measurement of these faster oscillations has been technically demanding, associations of beat-to-beat BPV with demographic, clinical, or psychosocial variables linked to health have been described only in a few small studies but have never been comprehensively evaluated in a large community-based sample. Here, we report on characteristics associated with resting beat-to-beat BPV measured in the low frequency domain using data from the multi-center MIDUS (Midlife in the US) study.

## Materials and methods

### Participants

MIDUS is a longitudinal cohort study of the behavioral, psychological and social factors accounting for age-related variation in health and wellbeing in a national sample of young to older age Americans (Brim et al., 2004). Data for the current study are from MIDUS 2 (M2), conducted between 2004 and 2006, a 9-year follow-up of the MIDUS 1 cohort, and from the MIDUS Refresher (MR), conducted between 2011 and 2014. M2 and MR consisted of five projects, including a Biomarker Substudy of 1,255 M2 and 863 MR participants, with data collection conducted during 1.5-day visits to a clinical research center (CRC) at the University of Wisconsin, Madison, University of California, Los Angeles, or Georgetown University. M2 Biomarker data were collected from mid-2004 to mid-2009. MR Biomarker data were collected from 2012 to 2016. IRB approval was obtained for data collection at the three sites and written consent was obtained from all study participants.

### Demographic characteristics

Demographic characteristics (age, race, sex, educational attainment, and household income) were collected by telephone and mail surveys. Household income is expressed as a ratio of total household income to the federal poverty level for a household of the size indicated by the participant.

### Physical examination

Clinicians or trained staff evaluated vital signs, morphology, functional capacities, bone densitometry and medication usage and performed a physical exam. A medical history was obtained, and a health condition burden score was calculated as the total number of self-reported experiences of eighteen health conditions (diabetes, cancer, heart disease, hypertension, circulation problems, blood clots, stroke/transient ischemic attack (TIA), lung problems, arthritis, thyroid disease, heart murmur, anemia or other blood disease, peptic ulcer disease, alcoholism, depression, cirrhosis/liver disease, cholesterol problems, eye disease). Body mass index (BMI) was assessed via measured height and weight during the physical exam. Participants were asked to bring all prescribed medications to the clinic visit to record the name and dosage. They were asked to indicate the reason for taking each medication as well as to report the use of any medications they did not bring to the clinic visit.

### Psychophysiological protocol

After an overnight stay at the CRC, participants were given a light breakfast, but no caffeine consumption was permitted. Following breakfast, they began a psychophysiology protocol. The finger cuff of a Finometer beat-to-beat blood pressure monitor (Finapres Medical Systems, Amsterdam, Netherlands) was placed around the middle finger of the non-dominant hand. ECG electrodes were placed on the left and right shoulders as well as in the left lower quadrant. Respiration was measured using an Inductotrace respiration monitor (Ambulatory Monitoring Systems, Ardsley, NY). Respiration bands were placed around the chest and abdomen and the respiration signal was calibrated using an 800 cc spirobag.

While participants were in the seated position, data were recorded during an 11-min baseline, exposure to two 6-min psychological challenges (mental arithmetic and the Stroop color-word matching task), 6-min recovery periods following each challenge, and a 6-min orthostatic challenge. The order of the psychological challenges was counterbalanced. Here we report data from the 11-min resting baseline.

### Collection of physiological signals

Analog ECG and BP signals were digitized at 500 Hz by a 16-bit A/D conversion board (National Instruments, Austin, TX) and passed to a microcomputer. Respiration signals were collected at 20 Hz. The ECG and BP waveforms were submitted to custom-written software that detected the time of each R wave and the time and magnitude of each systolic peak and diastolic trough, resulting in RR interval (RRI) and BP time series. Errors in marking R waves and systolic and diastolic values were identified by visual inspection. Values corresponding to ectopic beats were corrected by interpolation. Signals from thoracic and abdominal stretch bands were submitted to the same software to compute respiratory rate on a minute-by-minute basis.

LF-BPV (0.04–0.15 Hz) was computed based on 300-s epochs, using an interval method for computing Fourier transforms similar to that described by DeBoer, Karemaker and Strackee (DeBoer et al., 1984). Prior to computing Fourier transforms, the mean of the BP series was subtracted from each value in the series. The series was filtered using a Hanning window (Harris, 1978) and the power over the LF band was summed. Estimates of spectral power were adjusted to account for attenuation produced by this filter (Harris, 1978). BPV data were computed only during periods in which the respiratory rate was above the LF band (7 breaths/min).

#### Biomarkers of risk

Details of biomarker collection appear in a supplement to (Gruenewald et al., 2012). Blood was collected during a fasting morning collection at the clinic. After centrifuging and aliquoting, samples were either frozen (for assays of cholesterol biomarkers, inflammatory biomarkers, and serum dehydroepiandrosterone sulfate) or refrigerated (for assay of glycosylated hemoglobin) until they were shipped to the MIDUS Biocore Lab at the University of Wisconsin for assay processing.

Urine was collected during a 12-h overnight protocol, from 7:00 pm to 7:00 am. Participants were instructed to discard the initial void and then to collect all subsequent voids in a collection container. Containers were refrigerated during the 12-h overnight collection period, after which they were sent to the lab for aliquoting into two test tubes to store urine for later assays of catecholamines and cortisol. The tubes for catecholamine assays contained acetic acid to acidify the urine. Acetic acid was not added to the tubes for the cortisol assay. Samples were then frozen (−60°C to −80°C) for later assay.

#### Mortality

In M2 participants only, mortality follow-up from various sources was conducted through 2021. All known or suspected decedents were submitted to a National Death Index (NDI) Plus search through 2021 (N = 1,382), the most recent year NDI offered complete records. These data were confirmed or supplemented with tracing and mortality closeout interviews conducted by the University of Wisconsin Survey Center and through longitudinal sample maintenance conducted by the MIDUS Administrative Core (N = 131). Cause of death information was obtained from the NDI Plus search, supplemented by other sources if NDI matches were unavailable.

#### Sample weights

Sample weights provided by the MIDUS 2 and MIDUS Refresher study were included in analyses utilizing sociodemographic data to compute estimates representative of the target population. Doing so reduces bias in our parameter estimates and allows appropriate inferences that can be generalized to the larger population of interest, and not just our unique sample (Bell et al., 2012). For these weighted analyses, only participants with available weights were analyzed (*n* = 636 from MIDUS 2 and *n* = 743 from MIDUS refresher). These participants were obtained through Random Digit Dialing and were not identified as partial replicates which removed twins, siblings, and city-oversampled participants. Several MIDUS sample weights were generated post-stratification and for this analysis, weights that adjusted for race, age, and education were used to approximate the target population derived from the Current Population Survey from the United States Census Bureau: October 2005 for MIDUS 2 and October 2012 for MIDUS refresher (Ryff et al., 2017). In weighted analyses, the sample weights were rescaled to match our observed total sample size using a simple weight calibration method thus allowing an interpretable estimate of the total weighted sample. Rescaling has no effect on computed estimates, such as means and proportions, since all weights are multiplied by the same scalar value (Brick and Kalton, 1996). SAS survey procedures were used to obtain appropriate standard errors. Weights were only utilized in analyses for which inferences to the larger population were planned.

### Statistical analysis

Prior to analysis, all variables were examined for distribution and outliers. Baseline characteristics were summarized by means and standard deviations for continuous variables and frequencies and proportions for categorical variables. Due to the skewed distributions of the blood pressure variability (LF-SBPV, LF-DBPV), a natural log transformation was applied to these variables prior to analysis.

#### Associations between physiological/sociodemographic characteristics and BP, BPV

A series of three linear regression models were fit for each of the outcomes (BP: systolic BP, diastolic BP; BPV: systolic LF-BPV, diastolic LF-BPV) to assess the association between physiological measures and sociodemographic characteristics. Model 1 assessed the single-predictor relationship of each BP and BPV measure during the baseline period with each demographic characteristic (age, sex, race, education level, and income level). Model 2 assessed the relationship of each BPV outcome to each sociodemographic characteristic while controlling for the baseline value of the corresponding baseline BP measure (i.e., LF-SBPV was adjusted for SBP, LF-DBPV for DBP). Model 3 assessed the relationship of each BPV outcome with each sociodemographic characteristic while controlling for the baseline value of the corresponding BP measure, all other sociodemographic characteristics, as well as medication usage and the presence or absence of the following disease conditions: history of heart disease, hypertension, diabetes, stroke, TIA, hypercholesterolemia, COPD, thyroid disease, vascular disease, asthma, smoking status, or depression. Medication covariates included major medications with known cardiovascular, autonomic, or neurologic effects.

Weights were utilized in all models assessing the relationship of the BP and BPV physiological measures with the sociodemographic characteristics.

#### Associations of baseline LF-BPV with biomarkers and biological health conditions

To explore associations with biological health conditions, resting/baseline BP and BPV were summarized using means and SDs for each of the following health conditions: hypertension, hypotension, diabetes, elevated CRP, obesity, cholesterol, LDL, HDL, and hypertriglyceridemia using validated diagnostic cutoffs. Cutoffs used were: ≥140 mmHg SBP or ≥90 mmHg DBP for hypertension; ≤60 mmHg DBP for hypotension; HbA1c ≥6.5% for Diabetes; CRP ≥3 mg/L for elevated CRP; BMI≥30 kg/m^2^ for obesity; cholesterol ≥200 mg/dL, LDL ≥100 mg/dL, HDL <60 mg/dL, and triglycerides >200 mg/dL for hypertriglyceridemia.

To examine whether BPV was associated with these validated biological health conditions, after controlling for BP, a series of logistic regression models were performed: Model 1 estimated the odds of each biological health condition while controlling for BP and covariates of age, sex, race, education, and income; Model 2 included the same effects as Model 1, but additionally controlled for BPV. Significant BPV effects in Model 2, when adjusted for BP, indicates that the combination of BPV and BP explain more of the variance in the outcome than BP alone. These models were also repeated with additional adjustment for any comorbidity (single variable; “any of selected comorbidities” vs. “none of the selected comorbidities”) and medication covariates (single variable; “any of selected medications” vs. “none of the selected medications”) (Models 3 & 4).

Linear regression models were used to assess whether LF-BPV significantly explained additional variability in the biomarker outcomes beyond that explained by BP and covariates alone. Coefficient of determination (*R* ^2^) values were computed and then compared between nested models that first included BP and covariates (Model 1) and then additionally included BPV (Model 2). As with logistic regression models described above, linear models were also then repeated with adjustment for any comorbidity and medication covariates (Models 3 & 4).

#### Associations between physiological measures and mortality

Cox proportional hazards models were fit to assess the effect of BPV on mortality risk, separately for systolic and diastolic LF-BPV, while adjusting for the corresponding BP value. Models were then repeated, adjusting, in separate models, for age, race, and education.

All analyses were carried out using SAS^®^ version 9.4. All statistical tests were two-sided and used a *p* < 0.05 to determine statistical significance.

## Results

### Demographic characteristics

Weighted and unweighted sociodemographic characteristics of the combined M2 and MR samples are presented in Table 1. Participants in the unweighted sample (N = 2,118) were on average 54.7 (standard deviation (SD) = 12.7) years of age; 54.9% were women, 75.4% white people, 17.8% black people, and 6.7% were from other or mixed racial groups. Participants were fairly well-educated, although about 23.6% had only a high school degree or less. The average household income to poverty ratio also fell in the higher range but about 20.1% of those who reported household income reported incomes at less than 200% of the federal poverty level, which is the threshold to qualify for financial and other forms of assistance in some states. Nearly half the sample did not have reported household income, however, and it cannot be determined if the unreported income levels would be similarly distributed. About a quarter of the sample reported having no significant health conditions (25.6%) and reported taking no medications that affected the activity of the cardiovascular and autonomic nervous systems (26.8%). Participants were on average at the bottom of the obesity range (mean BMI = 30.0 kg/m^2^, SD = 7.1. Characteristics of the weighted sample were not meaningfully different.

**TABLE 1:** Weighted and unweighted sociodemographic characteristics of the MIDUS 2 and MIDUS refresher biomarker samples.

		Unweighted sample		Weighted sample	
Measures	Level	N	Mean (SD) or %	N	Weighted mean (SD) or %
Survey sample	MIDUS-2	1,255	59.25	636	41.42
	MIDUS-R	863	40.75	746	58.58
Sociodemographics					
Age	years	2,118	54.68 (12.74)	1,382	52.78 (12.87)
Age group	Younger (25–49)	756	35.69	481	40.13
	Middle-aged (50–64)	878	41.45	549	41.21
	Older (65–86)	484	22.85	352	18.66
Sex	Male	955	45.09	674	47.64
	Female	1,163	54.91	708	52.36
Race	White	1,591	75.44	1,184	83.22
	African-American	376	17.83	75	8.76
	Other race	142	6.7	117	7.66
	(missing)	9		6	0.36
Education	HS/GED or less	499	23.60	262	30.50
	AA degree/some college	638	30.18	400	28.11
	BA degree or higher	977	46.22	719	41.28
	(missing)	4		1	0.11
HH income to poverty ratio	<200% FPL	231	20.12	111	7.49
	200-<400% FPL	261	22.74	146	9.32
	400-<600% FPL	259	22.56	141	9.97
	>= 600% FPL	397	34.58	225	13.79
	(missing)	970		759	59.42
Any comorbidity^1^	No	542	25.59	353	26.09
	Yes	1,576	74.41	1,029	73.91
Medication use	No	567	26.77	329	24.46
	Yes	1,551	73.23	1,053	75.54
BMI	kg/m^2^	2,117	30.03 (7.07)	1,381	30.25 (7.15)
Physiology					
Systolic BP	mmHg	1813	123.84 (18.36)	1,173	123.17 (16.91)
Diastolic BP	mmHg	1813	61.97 (11.65)	1,173	62.34 (10.62)
ln (Systolic LF-BPV)	ln (mmHg^2^)	1813	2.26 (0.80)	1,173	2.25 (0.73)
ln (Diastolic LF-BPV)	ln (mmHg^2^)	1813	1.05 (0.81)	1,173	1.06 (0.73)

### Demographic variations in resting BP and BPV

Mean levels of SBP, DBP, SBPV and DBPV are reported in Table 1. Mean levels by demographic characteristics for the weighted sample appear in Table 2 (model 1, ANOVA), along with two sets of covariate-adjusted means (model 2 & 3, ANCOVA).

**TABLE 2:** Weighted means (SE) and covariate-adjusted means (SE) of BP and BPV during seated rest by demographic characteristics.

	SBP			DBP			ln LF-SBPV			ln LF-DBPV		
	M1	M2	M3	M1	M2	M3	M1	M2	M3	M1	M2	M3
Seated baseline	Mean (SE)		Mean (SE)	Mean (SE)		Mean (SE)	Mean (SE)	Mean (SE)	Mean (SE)	Mean (SE)	Mean (SE)	Mean (SE)
Age group												
Younger (25–49)	122.77 (0.92)	NA	123.48 (0.95)	65.02 (0.54)	NA	65.08 (0.59)	2.28 (0.04)	2.29 (0.04)	2.29 (0.04)	1.35 (0.03)	1.33 (0.03)	1.31 (0.03)
Middle-aged (50–64)	122.43 (0.92)	NA	122.55 (0.88)	61.31 (0.57)	NA	61.38 (0.56)	2.28 (0.04)	2.29 (0.04)	2.28 (0.04)	0.94 (0.04)	0.95 (0.04)	0.95 (0.04)
Older (65–86)	126.19 (1.36)	NA	124.95 (1.35)	57.68 (0.87)	NA	57.57 (0.87)	2.07 (0.06)	2.06 (0.06)	2.07 (0.06)	0.57 (0.06)	0.60 (0.06)	0.65 (0.06)
*P*	0.0573	NA	0.3180	**<.0001**	NA	**<.0001**	**0.0070**	**0.0013**	**0.0054**	**<.0001**	**<.0001**	**<.0001**
Sex												
Male	128.35 (0.84)	NA	128.62 (0.87)	64.35 (0.52)	NA	64.46 (0.54)	2.37 (0.04)	2.35 (0.04)	2.34 (0.04)	1.13 (0.04)	1.10 (0.04)	1.12 (0.04)
Female	118.67 (0.77)	NA	118.58 (0.74)	60.60 (0.50)	NA	60.05 (0.47)	2.13 (0.04)	2.17 (0.04)	2.17 (0.04)	1.00 (0.04)	1.02 (0.04)	1.01 (0.03)
*P*	**<.0001**	NA	**<.0001**	**<.0001**	NA	**<.0001**	**<.0001**	**0.0007**	**0.0018**	**0.0121**	0.1179	**0.0258**
Race												
White	123.31 (0.64)	NA	123.20 (0.61)	62.18 (0.38)	NA	62.04 (0.36)	2.27 (0.03)	2.27 (0.03)	2.27 (0.03)	1.06 (0.03)	1.06 (0.03)	1.07 (0.03)
African-American	124.41 (2.43)	NA	127.29 (2.45)	65.57 (1.88)	NA	65.83 (1.96)	2.03 (0.12)	2.03 (0.12)	2.07 (0.12)	0.94 (0.13)	0.89 (0.13)	0.92 (0.12)
Other race	120.23 (1.84)	NA	120.73 (1.82)	60.24 (1.02)	NA	59.18 (0.98)	2.26 (0.09)	2.29 (0.09)	2.25 (0.08)	1.17 (0.08)	1.20 (0.08)	1.07 (0.07)
*P*	0.2447	NA	0.0996	**0.0335**	NA	**0.0022**	0.1529	0.1436	0.2831	0.2787	0.1112	0.4471
Education												
HS/GED or less	122.56 (1.38)	NA	122.67 (1.34)	61.78 (0.80)	NA	61.98 (0.78)	2.18 (0.06)	2.19 (0.06)	2.22 (0.06)	0.95 (0.06)	0.95 (0.06)	1.00 (0.05)
AA degree/some college	122.86 (1.01)	NA	122.91 (0.95)	62.28 (0.65)	NA	61.94 (0.61)	2.26 (0.05)	2.27 (0.05)	2.26 (0.05)	1.07 (0.05)	1.07 (0.05)	1.06 (0.05)
BA degree or higher	123.81 (0.74)	NA	124.21 (0.76)	62.78 (0.49)	NA	62.43 (0.50)	2.28 (0.03)	2.28 (0.03)	2.26 (0.03)	1.14 (0.03)	1.13 (0.03)	1.10 (0.03)
*P*	0.6235	NA	0.4522	0.5441	NA	0.7907	0.3277	0.3932	0.8307	**0.0189**	**0.0301**	0.2964
HH income to poverty ratio												
<200% FPL	123.52 (1.07)	NA	124.22 (0.98)	62.02 (0.61)	NA	62.30 (0.61)	2.22 (0.05)	2.23 (0.05)	2.25 (0.05)	1.01 (0.05)	1.01 (0.05)	1.03 (0.05)
200-<400% FPL	124.93 (2.42)	NA	124.58 (2.34)	62.95 (1.66)	NA	62.71 (1.59)	2.27 (0.11)	2.26 (0.11)	2.24 (0.11)	1.09 (0.10)	1.08 (0.11)	1.11 (0.10)
400-<600% FPL	123.45 (2.29)	NA	122.17 (2.35)	62.72 (1.34)	NA	61.41 (1.49)	2.25 (0.10)	2.26 (0.09)	2.20 (0.09)	1.10 (0.10)	1.09 (0.10)	1.04 (0.09)
>= 600% FPL	120.31 (2.12)	NA	119.32 (2.09)	63.16 (1.24)	NA	61.60 (1.27)	2.34 (0.09)	2.37 (0.09)	2.29 (0.09)	1.27 (0.09)	1.25 (0.09)	1.16 (0.08)
*P*	0.2448	NA	0.1086	0.9174	NA	0.8752	0.6593	0.4424	0.7183	**0.0443**	0.0631	0.4304

M1—Model 1 regressions featured demographic variables individually.

M2—Model 2 added SBP, for SBPV, and DBP, for DBPV, as predictors (No model 2 was run for SBP, and DBP, as outcomes).

M3—Model 3 regressions featured all demographic predictors jointly, as well as the corresponding BP, variable for BPV, and 3 additional binary covariates (Health condition, medication use, smoking status).

Means and SEs, are presented for each level of the predictors, as well as *p*-values for each predictor’s overall effect.

The bold value means significant at < 0.05.

#### Age

The unadjusted mean levels of DBP were significantly lower in the older age groups (*p* < .0001). In model 3, after control for covariates, the association of age with DBP remained highly significant. Age was significantly and negatively associated with both LF-SBPV and LF-DBPV in all models.

#### Sex

In both the unadjusted and fully adjusted models, BP and LF-BPV differed by sex, with males having significantly higher means for all parameters.

#### Race

There was no association between race and SBP, but in both the unadjusted and fully adjusted models, DBP was greater in black participants compared to the other racial groups. LF-BPV was not significantly associated with race.

#### Education

SBP, DBP, or LF-SBPV were not significantly associated with educational attainment. Educational attainment was positively and significantly associated with LF-DBPV in the unadjusted model but not in the fully adjusted model.

#### Income

Income was not associated with SBP, DBP, or LF-SBPV in neither the unadjusted nor adjusted models. LF-DBPV was significantly and positively associated with income in the unadjusted model but not in the adjusted model.

### Relationship of health outcomes to resting BPV

Tables 3–5 present results from logistic regression models estimating the odds of health outcomes, defined using diagnostic thresholds for continuous biomarkers. Model 1 estimates the effects of BP (either systolic or diastolic) on the odds of health outcomes with covariates age, sex, race, education, income, and smoking status. Model 2 features the same set of predictors while adding ln LF-BPV (again either systolic or diastolic). Tests of the BP effect, in both models, and the ln LF-BPV effect in Model 2 are presented. Models 3 and 4 repeat the analysis additionally adjusting for any comorbidity and medication covariates. For hypertension/hypotension models, BP was not included as a predictor as it was used to define the outcome.

**TABLE 3:** Logistic regression model results of BP and BPV estimating hypertension and hypotension unweighted data.

	Adjusted for age, sex, race, education, income, and smoking status						Additionally adjusted for health conditions and meds covariates					
	Model 1		Model 2				Model 3		Model 4			
	BP		BP		BPV		BP		BP		BPV	
Heath outcome	Est (log-odds)	p	Est (log-odds)	p	Est (log-odds)	p	Est (log-odds)	p	Est (log-odds)	p	Est (log-odds)	p
Seated baseline												
Hypertension (S) (SBP>=140)												
Systolic^a^	—	—	—	—	0.342	**<.001**	—	—	—	—	0.351	**<.001**
Diastolic	0.154	**<.001**	0.154	**<.001**	0.067	0.508	0.155	**<.001**	0.154	**<.001**	0.091	0.374
Hypertension (D) (DBP>=90)												
Systolic	0.109	**<.001**	0.109	**<.001**	0.034	0.906	0.111	**<.001**	0.110	**<.001**	0.093	0.756
Diastolic^a^	—	—	—	—	0.544	**0.046**	—	—	—	—	0.614	**0.026**
Hypertension (Either or both)												
Systolic^a^	—	—	—	—	0.336	**<.001**	—	—	—	—	0.346	**<.001**
Diastolic^a^	—	—	—	—	0.298	**<.001**	—	—	—	—	0.323	**<.001**
Hypotension (DBP<=60)												
Systolic	−0.089	**<.001**	−0.091	**<.001**	0.259	**<.001**	−0.089	**<.001**	−0.091	**<.001**	0.264	**<.001**
Diastolic^a^	—	—	—	—	−0.266	**<.001**	—	—	—	—	−0.280	**<.001**

^a^ Models do not control for the respective BP, that defines the outcome which leads to perfect AUC.

The bold value means significant at < 0.05.

**TABLE 4:** Logistic regression model results of BP and BPV estimating diabetes, obesity, and CRP unweighted data.

	Adjusted for age, sex, race, education, income, and smoking status						Additionally adjusted for health conditions and Meds covariates					
	Model 1		Model 2				Model 3		Model 4			
	BP		BP		BPV		BP		BP		BPV	
Heath outcome	Est (log-odds)	p	Est (log-odds)	p	Est (log-odds)	p	Est (log-odds)	p	Est (log-odds)	p	Est (log-odds)	p
Seated baseline												
Diabetes (HBA1C>=6.5)												
Systolic	0.006	0.116	0.006	0.154	0.063	0.505	0.006	0.134	0.005	0.233	0.145	0.130
Diastolic	−0.015	**0.030**	−0.013	0.068	−0.225	**0.020**	−0.014	**0.037**	−0.013	0.056	−0.108	0.273
Elevated CRP (CRP>=3 mg/L)												
Systolic	0.008	**0.006**	0.010	**0.002**	−0.174	**0.013**	0.008	**0.009**	0.009	**0.003**	−0.150	**0.033**
Diastolic	0.009	0.066	0.011	**0.022**	−0.256	**<.001**	0.009	0.076	0.011	**0.030**	−0.213	**0.005**
Obesity (BMI>=30)												
Systolic	0.014	**<.001**	0.015	**<.001**	−0.161	**0.012**	0.013	**<.001**	0.014	**<.001**	−0.120	0.065
Diastolic	0.007	0.127	0.009	**0.040**	−0.266	**<.001**	0.007	0.132	0.009	0.060	−0.191	**0.006**

The bold value means significant at < 0.05.

**TABLE 5:** Logistic regression model results of BP and BPV estimating metabolic alterations (Cholesterol & triglycerides) unweighted data.

	Adjusted for age, sex, race, education, income, and smoking status						Additionally adjusted for health conditions and Meds covariates					
	Model 1		Model 2				Model 3		Model 4			
	BP		BP		BPV		BP		BP		BPV	
Heath outcome	Est (log-odds)	p	Est (log-odds)	p	Est (log-odds)	p	Est (log-odds)	p	Est (log-odds)	p	Est (log-odds)	p
Seated baseline												
Hypertriglyceridemia (Triglycerides>=200)												
Systolic	0.001	0.873	−0.000	0.993	0.093	0.332	0.000	0.948	−0.001	0.889	0.112	0.243
Diastolic	−0.016	**0.017**	−0.016	**0.019**	−0.014	0.884	−0.016	**0.019**	−0.016	**0.019**	0.019	0.848
High cholesterol (Cholesterol>=200)												
Systolic	0.004	0.179	0.003	0.288	0.104	0.125	0.004	0.180	0.003	0.277	0.092	0.174
Diastolic	0.012	**0.009**	0.011	**0.018**	0.124	0.083	0.013	**0.005**	0.012	**0.010**	0.110	0.131
High LDL (LDL>=100)												
Systolic	0.003	0.214	0.003	0.355	0.118	0.062	0.004	0.185	0.003	0.298	0.100	0.115
Diastolic	0.014	**0.002**	0.013	**0.004**	0.123	0.064	0.014	**0.001**	0.014	**0.002**	0.095	0.160
Low HDL (HDL<60)												
Systolic	0.002	0.550	0.001	0.636	0.046	0.486	0.001	0.644	0.001	0.788	0.074	0.274
Diastolic	−0.002	0.594	−0.002	0.628	−0.023	0.741	−0.003	0.552	−0.003	0.526	0.024	0.740

The bold value means significant at < 0.05.

For systolic hypertension in Model 2, there was a significant positive effect of systolic ln LF-SBPV (Est = 0.342; *p*<=0.001). Similar results were found for any (systolic or diastolic) hypertension for both LF-SBPV (Est = 0.336; *p*<=0.001) and LF-DBPV (Est = 0.298; *p* < 0.001). For diastolic hypertension, diastolic ln LF-BPV was found to be significantly positively associated (Est = 0.544; *p* = 0.046), while for diastolic hypotension, an negative association was found (Est = −0.266; *p* < 0.001). Results were qualitatively the same for models adjusting for any comorbidity and medication variables. Ln LF-BPV did not significantly improve the model of the odds of the other health outcomes.

### BP and LF-BPV at rest and biomarkers

Table 6 presents coefficients of determination (*R* ^2^) for linear regression models modeling continuous biomarkers. Model 1 *R* ^2^ estimates the proportion of variance in the biomarker outcome that can be accounted for by BP (either systolic or diastolic) and covariates age, sex, race, education, income, and smoking status. Model 2 *R* ^2^ estimates the proportion of variance that can be accounted for by the same set of predictors plus ln LF-BPV (again either systolic or diastolic). Models 3 and 4 repeat the analysis further adjusting for any comorbidity and medication covariates.

**TABLE 6:** Linear regression models modeling the association of biomarkers with BP and BPV unweighted data.

	Adjusted for age, sex, race, education, income, and smoking status								Additionally adjusted for any comorbidity and any medication							
	Model 1			Model 2					Model 3			Model 4				
Biomarker	R^2^	BP	BP p	R^2^	BP	BP p	BPV	BPV p	R^2^	BP	BP p	R^2^	BP	BP p	BPV	BPV p
		Est			Est		Est			Est			Est		Est	
Seated baseline																
ln IL-6																
Systolic	0.14	0.003	**<.001**	0.14	0.004	**<.001**	−0.035	0.104	0.15	0.003	**<.001**	0.16	0.003	**<.001**	−0.024	0.254
Diastolic	0.13	0.002	0.211	0.14	0.003	0.069	−0.095	**<.001**	0.15	0.002	0.205	0.16	0.003	0.084	−0.076	**<.001**
ln e-Selectin																
Systolic	0.06	0.002	**0.018**	0.06	0.002	**0.022**	0.004	0.776	0.07	0.001	**0.025**	0.07	0.001	**0.036**	0.010	0.510
Diastolic	0.06	0.002	**0.026**	0.06	0.003	**0.018**	−0.020	0.215	0.07	0.002	**0.028**	0.07	0.002	**0.024**	−0.009	0.555
ln s-ICAM																
Systolic	0.06	0.000	0.703	0.06	0.000	0.489	−0.023	0.059	0.06	0.000	0.751	0.06	0.000	0.542	−0.022	0.081
Diastolic	0.06	0.001	0.213	0.06	0.001	0.116	−0.032	**0.014**	0.06	0.001	0.211	0.06	0.001	0.120	−0.029	**0.027**
ln Epinephrine																
Systolic	0.39	−0.002	0.386	0.40	−0.002	0.252	0.076	0.080	0.40	−0.002	0.356	0.40	−0.002	0.211	0.088	**0.043**
Diastolic	0.39	0.000	0.952	0.39	−0.001	0.862	0.085	0.066	0.40	−0.000	0.958	0.40	−0.001	0.724	0.106	**0.023**
ln Norepinephrine																
Systolic	0.39	0.002	0.085	0.39	0.002	0.139	0.044	0.181	0.39	0.002	0.103	0.39	0.002	0.185	0.056	0.089
Diastolic	0.39	0.004	0.082	0.39	0.004	0.113	0.036	0.305	0.39	0.004	0.098	0.39	0.003	0.158	0.058	0.098
ln Cortisol																
Systolic	0.15	0.001	0.617	0.15	0.000	0.923	0.062	**0.014**	0.16	0.001	0.523	0.17	0.000	0.764	0.050	**0.045**
Diastolic	0.15	−0.000	0.896	0.15	−0.001	0.607	0.075	**0.005**	0.16	−0.000	0.973	0.16	−0.001	0.750	0.054	**0.043**

The bold value means significant at < 0.05.

#### IL-6

During seated rest, SBP was significantly and positively related to IL-6, a relationship that remained significant when LF-SBPV was added to the model. LF-SBPV did not significantly improve the model. DBP was not related to IL-6 either before or after LF-DBPV was added, but LF-DBPV was significantly and negatively related to IL-6 in Models 2 and 4.

#### E-selectin

Both SBP and DBP were significantly and positively related to E-selectin before and after adjusting for LF-SBPV and -DBPV in the model. Neither measure of BPV was significantly related to e-selectin with BP in the model.

#### s-ICAM

Neither SBP nor DBP was related to s-ICAM before or after including LF-BPV in the models. With BP in the model, LF-SBPV and -DBPV were negatively and marginally (LF-SBPV) or significantly (LF-DBPV) related to s-ICAM.

#### Epinephrine

Neither SBP nor DBP was related to epinephrine before or after including LF-BPV in the models. With BP in the model, LF-SBPV and -DBPV were positively related to epinephrine, though only when also adjusted for any comorbidity and any medication.

#### Norepinephrine

Neither SBP nor DBP was related to norepinephrine before or after including LF-BPV in the models. With BP in the model, neither LF-SBPV nor -DBPV was positively related to norepinephrine.

#### Cortisol

Neither SBP nor DBP was related to cortisol before or after including LF-BPV in the models. With BP in the model, LF-SBPV and -DBPV were positively related to cortisol before and after adjusting any comorbidity and any medication usage.

#### BPV and mortality

Because HRV has been shown to predict morbidity and mortality in both clinical (Kleiger et al., 1987; Bigger et al., 1992; La Rovere et al., 1998) and community samples (Tsuji et al., 1996; Liao et al., 1997; Liao et al., 2002), we also sought to determine whether resting LF-BPV was associated mortality. Of 1255 MIDUS 2 participants, 217 died during follow-up (11.1 ± 1.2 years). In Table 7, we present hazard ratios (with 95% CIs) and *p*-values from Cox proportional-hazard models estimating mortality from ln LF-BPV. Models were run adjusted only for corresponding BP (systolic or diastolic), and then re-run adjusting, one-by-one, for age, race, and education, in separate models. Resting systolic LF-SBPV was unrelated to mortality in all models. However, diastolic LF-BPV was significantly negatively related to mortality, with one unit higher ln LF-DBPV associated with a 21.8% lower mortality hazard during the follow-up period (HR = 0.792; 95% CI = 0.652, 0.962; *p* = .0010). This association remained significant with adjustment for race, education, but not when adjusted for age (HR = 0.954; 95% CI = 0.781, 1.164; *p* = .6404), suggesting that age may confound the association between LF-DBPV and mortality.

**TABLE 7:** Association between LF-BPV and mortality.

	Unadjusted		Adjusted by age		Adjusted by education		Adjusted by race	
Predictor	Hazard ratios (95% CI)	*p*-value	Hazard ratios (95% CI)	*p*-value	Hazard ratios (95% CI)	*p*-value	Hazard ratios (95% CI)	*p*-value
Systolic BP variation	0.963 (0.787,1.179)	0.7158	1.086 (0.886,1.330)^b^	0.4274	0.968 (0.789,1.187)	0.7551	0.963 (0.788,1.178)	0.7163
Diastolic BP variation	**0.792 (0.652,0.962)**	**0.0010**	0.954 (0.781,1.164)^c^	0.6404	**0.802 (0.660,0.976)**	**0.0274**	**0.797 (0.657,0.968)**	**0.0223**

^a^ Models were run separately for systolic and diastolic LF-BPV. Each adjusted model was adjusted by the specific covariate and systolic or diastolic LF-BPV.

Significant covariates:

^b^ Age was significantly associated with mortality in this model. Middle-aged subjects had 1.8 times the mortality hazard rate of young subjects (95% CI: 1.0, 3.0) and older subjects had 4.9 times the mortality hazard rate of young subjects (95% CI: 2.9, 8.2) during the observed period.

^c^ Age was significantly associated with mortality in this model. Older subjects had 4.1 times the mortality hazard rate of young subjects (95% CI: 2.4, 7.1) during the observed period.

The bold value means significant at < 0.05.

## Discussion

Variability in HR conveys significant prognostic information not only for a variety of patient groups but also for healthy individuals in community studies. In addition, it provides a window on the activity of the autonomic nervous system. These advances in HRV research have been possible because in clinical settings or even in the field, recording the continuous ECG signal, from which HRV is derived, is relatively simple.

Like heart rate, blood pressure is not steady and it varies across multiple time scales. VVV-BP over days, months, or even years has been associated with a variety of clinical outcomes including mortality. ABPV over 24-h periods similarly has been related to multiple clinical outcomes.

BP also varies on a beat-to-beat basis and it oscillates in the same low frequency range as HR. Advances in analysis of beat-to-beat BPV have lagged behind HRV because of technical limitations. Noninvasive acquisition and recording of the continuous BP signal, essential for studies with large samples, require devices like the Finometer (Finapres Medical Systems BV, Amsterdam, the Netherlands) or Nexfin (BMEYE, Amsterdam, Noord-Holland, Netherlands) that use the volume-clamp method.

Compared with ECG monitors, these devices are expensive and more complex to use. As a result, measurement of beat-to-beat BPV in clinical settings and community studies is relatively uncommon. We capitalized on the Biomarker project of the MIDUS study to record the continuous BP signal during 11 min of seated rest. From these BP waveforms, we created BP time series which were submitted to Fourier-based spectral analysis to estimate LF-BPV. These estimates were used 1) to examine the relationships between LF-BPV and sociodemographic variables, biomarkers of risk, and mortality and 2) to contrast them with previously reported associations of VVV-BP and ABPV with these outcomes.

### LF-BPV and sociodemographic variables

#### Age

Age was negatively related to both LF-SBPV and -DBPV in the weighted sample of 1,382 participants. Some studies with much smaller samples also report this same negative relationship (Veerman et al., 1994; Xing et al., 2017). In contrast, another small study reported that LF-BPV was greater in older compared to younger healthy adults (Kiviniemi et al., 2010). Still another reported no association with age (Shi et al., 2003).

The negative relationship between LF-BPV and age in the MIDUS data also contrasts with studies reporting direct relationships between age and BPV measured at longer time scales, i.e., VVV and 24-h ABPV. In the NHANES III (Muntner et al., 2011) and ALLHAT (Muntner et al., 2015b) studies and in data from the Women’s Health Initiative (Shimbo et al., 2012) and in the US Veterans Administration system (Gosmanova et al., 2016) and the Korean National Health system (Bae et al., 2019), VVV-SBP was positively associated with age.

Studies of age and ABPV show a similar direct relationship. ABPV-SBP and -DBP increased with age in a random population sample of 8,938 from multiple geographic regions (Hansen et al., 2010). ABPV was greater in older (age >80 years) than younger (age 61–79 years) elderly participants (Sakakura et al., 2007), in older than younger hypertensive patients (Cicconetti et al., 2000), and in 7,112 untreated hypertensive patients although only during daytime (Palatini et al., 2014). However, some studies fail to show this relationship. In 27,472 primary care patients throughout India, ABPV-SBP increased but ABPV-DBP decreased with age (Kaul et al., 2019) and were unrelated to age in other studies (Acharya et al., 1996; Khattar et al., 2001; Li et al., 2017).

#### Sex

In unadjusted and covariate-adjusted models, both LF-SBPV and -DBPV were greater in men than in women. These findings largely contrast with those from studies of VVV-SBP, which was greater in women than in men (Muntner et al., 2011; Muntner et al., 2015b; Tedla et al., 2017; Bae et al., 2019), but in data from the US Veterans Administration, VVV-SBP was greater in men than in women (Gosmanova et al., 2016).

Studies reporting relationships between ABPV and sex are more mixed. ABPV-SBP and -DBP were greater in men than in women in 8,938 participants (Hansen et al., 2010), in a longitudinal study of 641 young participants (Li et al., 2010), and in 1,133 young participants in two twin studies (Xu et al., 2013) but there was no sex difference in several small studies of hypertensive patients (Cicconetti et al., 2000; Li et al., 2017; Pucci et al., 2017). In 723 hypertensive patients, ABPV-SBP and -DBP were greater in women compared to men (Acharya et al., 1996). A meta-analysis of 10 cohorts with 17,312 hypertensive patients found no significant sex difference in ABPV-SBP and -DBP (Roush et al., 2015).

#### Race

Whites and non-whites did not differ in either measure of LF-BPV. In contrast, VVV was greater in non-Hispanic black compared to white people in NHANES III (Muntner et al., 2011), ALLHAT (Muntner et al., 2015b), Veterans Administration data (Gosmanova et al., 2016), and in the Women’s Health Initiative (Shimbo et al., 2012).

Few studies report on racial differences in ABPV. In youth and young adults, ABPV-SBP and -DBP were greater in black people compared to white people (Li et al., 2010; Muntner et al., 2015a). In contrast, there were no racial differences in ABPV-SBP or -DBP in 723 hypertensive patients (Acharya et al., 1996) or in 1,133 participants in a twin study (Xu et al., 2013).

#### SES

Socioeconomic status, measured either as educational attainment or income, generally was unrelated to LF-BPV. In contrast, VVV-SBP was greater in lower income participants (Gosmanova et al., 2016; Bae et al., 2019) and in participants with lower levels of educational attainment in ALLHAT (Muntner et al., 2015b) and the Women’s Health Initiative (Shimbo et al., 2012). SES measured as the educational level of the father of black and white youth and young adults was also negatively related to ABPV-SBP and -DBP (Li et al., 2010).

### LF-BPV, medical comorbidities, and biomarkers of risk

LF-SBPV was positively related to systolic hypertension. There was no association between LF-DBPV and diastolic hypertension. LF-DBPV was significantly and negatively associated with an increased likelihood of diastolic hypotension. Otherwise, beyond these few significant associations, LF-BPV was not related to the comorbid medical conditions we considered.

LF-DBPV was significantly and negatively associated with both IL-6 and s-ICAM but not e-Selectin with and without control of comorbidities and medications. After control for covariates, both LF-SBPV and -DBPV were significantly and positively associated with urinary epinephrine and cortisol but not norepinephrine.

VVV-SBP was positively related to SBP (Muntner et al., 2011; Shimbo et al., 2012; Wang et al., 2016; Tedla et al., 2017; Mancusi et al., 2021), to BMI (Li et al., 2010; Shimbo et al., 2012; Wang et al., 2016; Tedla et al., 2017; Mancusi et al., 2021), and to serum cholesterol levels in multiple studies (Muntner et al., 2011; Shimbo et al., 2012; Muntner et al., 2015b; Wang et al., 2016; Mancusi et al., 2021) and to triglycerides (Okada et al., 2012; Wang et al., 2016). VVV-SBP was positively related to serum CRP (Muntner et al., 2011; Wang et al., 2016; Tedla et al., 2017), diabetes (Muntner et al., 2011; Shimbo et al., 2012; Mancusi et al., 2021), and arterial stiffness (Okada et al., 2012; Gosmanova et al., 2016) and to the progression of arterial stiffness in 1152 MESA study participants not taking antihypertensive medications (Tedla et al., 2017) and in 3,994 participants in a prospective, community-based study of Chinese adults (Wang et al., 2016) and to the change in LVMI in 3,555 patients 90 months later in the Campania Salute Network registry (Mancusi et al., 2021) and in 2,400 participants in the CARDIA study after 25-year follow-up (Nwabuo et al., 2020).

Multiple studies report associations between ABPV and risk biomarkers but findings are mixed. ABPV-SBP and -DBP were greater in patients with diabetes (Hansen et al., 2010; Palatini et al., 2014) and positively associated with BMI (Lurbe et al., 2006; Hansen et al., 2010; Li et al., 2010; Abramson et al., 2011; Palatini et al., 2014), and negatively related to serum triglycerides, total, HDL-, and LDL-cholesterol in patients with known or suspected hypertension in one study (Li et al., 2017) but positively related to total cholesterol in another (Hansen et al., 2010). Associations with inflammatory markers also are mixed {Abramson et al., 2006 #19260} {Tatasciore et al., 2008 #15115} {Kim et al., 2008 #10236} {Schein et al., 2019 #19343}.

Both ABPV-SBP and -DBP were positively related to LVMI in 1,648 participants in the PAMELA study (Sega et al., 2002) but not in another (Woodiwiss et al., 2017).

### Beat-to-beat LF-BPV and mortality

Finally, we examined whether LF-BPV was associated with mortality. LF-DBPV but not -SBPV was significantly and negatively related to mortality up to 15 years of follow-up. This effect became nonsignificant when age was added to the model because age and LF-DBPV were negatively associated with each other.

These findings contrast to reports of direct relationships between VVV-BP and ABPV and mortality in NHANES-III after 14 years of follow-up (Muntner et al., 2011), in MESA after 14.2 years of follow-up (de Havenon et al., 2021), in 33,357 hypertensive patients randomized to receive three different treatments in ALLHAT, VVV-SBP and VVV-DBP were positively associated with all-cause mortality after 2.8 years of follow-up (Muntner et al., 2015b), and in 2,865,157 patients with chronic kidney disease from US Department of Veterans Affairs facilities after 8 years of follow-up (Gosmanova et al., 2016). Systematic data analyses concluded that VVV-SBP predicted all-cause mortality (Tai et al., 2015; Stevens et al., 2016).

24-h ABPV-SBP and -DBP predicted all-cause mortality in 7,112 untreated hypertensive patients after follow-up of 5.5 years (Palatini et al., 2014), in 9,154 patients assessed for hypertension after a median follow-up of 6.3 years (Bilo et al., 2020), and in 8,938 patients with diabetes followed for a median of 11.3 years (Hansen et al., 2010). ABPV-DBP predicted all-cause mortality in untreated hypertensive but not normotensive community-based participants after 20 years of follow-up (Hsu et al., 2016). A systematic data analysis concluded that ambulatory SBPV predicted all-cause mortality (Stevens et al., 2016).

### Beat-to-Beat LF-BPV, VVV, and ABPV

To summarize, evidence suggests that spectrally defined LF-BPV derived from continuous BP recordings behaves differently with respect to sociodemographic characteristics, some risk biomarkers and medical comorbidities, and all-cause mortality compared to BPV measured at longer time scales, i.e., VVV-BP and ABPV. In most cases, relationships between VVV-BP and ABPV and these variables have been direct: greater BPV using either of these metrics is associated with greater levels of the outcome variables. In contrast, LF-BPV was negatively related to many of these outcomes.

Multiple factors may contribute to these differences. First, LF-BPV reflects regularly occurring oscillations in beat-to-beat BP only in the 0.04–0.15 Hz range. In contrast, VVV-BP and ABPV are measured by global indices of variability, typically the standard deviation, which reflects all sources of variation.

Second, the measurement conditions of LF-BPV differ substantially from those of VVV-BP and ABPV. 24-h ABPV is subject to variation due to multiple sources that may differ from measurement to measurement over the course of 24 h: posture, interpersonal interactions, substances consumed, physical activity, and sleep states (awake vs. asleep). VVV is subject to variations in some of these, e.g., mood and daily stress. LF-BPV, in contrast, was measured solely in the seated position in a strictly controlled setting with no distractions and thus is not subject to the influence of any of these factors.

Third, differences also exist in the number of readings used to calculate variability. In most VVV studies, this number is small, ranging from as few as 3 (Muntner et al., 2011) to as many as 24 (Gosmanova et al., 2016). Studies of ABPV may have 40–96 readings over a 24-h period. Estimates of variability are more stable with greater numbers of measurements. Comparison to LF-BPV is complex because even though BP is measured on a beat-to-beat basis, LF-BPV is calculated on a varying number of beats because it is computed in the 0.04–0.15 Hz range, i.e., oscillations ranging from periods of 6.67–25 s. Arbitrarily choosing the middle of this frequency range, i.e., a period of 16 s, LF-BPV would be computed from 37.5 cycles during the 10 min of beat-to-beat data submitted to analysis.

Finally, there are considerable differences in the samples studied. Some were community samples while others were treatment studies. Among community samples, some selected only healthy participants while others did not. Treatment studies varied by disease as well as its stage. Despite these differences, studies of VVV-BP and ABPV generally are consistent in their findings that greater BPV is associated with greater age, female sex, black race, adverse disease outcomes, and mortality. The relationship between LF-BPV and these characteristics and outcomes is largely in the opposite direction: Negatively associated with age, lower in women, not different in black and white people, and negatively related to mortality.

These differences in relation to risk markers and clinical outcomes suggest different underlying physiologies which in turn may have implications for treatment. High BPV, measured as VVV-BP and ABPV, may reflect deleterious structural changes in the arterial wall that lead to reduced bioavailability of nitric oxide, impairing vasodilatory capacity, and greater arterial stiffness from proliferation of smooth muscle cells (Shimbo et al., 2012; Wang et al., 2016; Nwabuo et al., 2020). Consistent with this account, interest in BPV as a therapeutic target independent of mean BP has grown. In spontaneously hypertensive rats, anti-hypertensive treatment-related decreases in left ventricular and aortic hypertrophy were more closely associated with reduction in systolic SD-BPV than with mean BP (Xie et al., 2008). In a human treatment study of 577 hypertensive patients with random assignment to placebo, amlodipine, candesartan, and indapamide, all 3 active treatments reduced BP to a similar extent but only amlodipine reduced 24-h ABPV although associations of 24-h ABPV and clinical outcomes were not reported (Zhang et al., 2011). A more recent study also reported that calcium channel blockers were superior to angiotensin converting enzyme inhibitors in reducing ABPV independent of changes in mean pressure (Parati et al., 2018). Based on evidence such as this, Schillaci et al. (2011) editorially commented “BP variability reduction should be considered as a possible new target to explore by future intervention trials in hypertension” and lamented the absence of data on beat-to-beat BPV (*p*. 135).

Because studies of LF-BPV and clinical outcomes are limited and because our data suggest directionally opposite relationships between clinical risk markers and outcomes and LF-BPV, on the one hand, and VVV-BP and ABPV, on the other, it is unclear whether reduction of LF-BPV should be a therapeutic goal. It also is unclear which BPV time scale is associated with the greatest benefit or risk (Millar, 2020). Indeed, recent studies, primarily of neurodegenerative disorders, suggest that LF-BPV may have a protective effect, secondary to greater distribution of blood flow (i.e., perfusion across the tissue), protection of tissue oxygenation, and the clearance of cellular and metabolic debris from interstitial fluid. Induced 0.1 Hz BP oscillations increased tolerance to hypovolemic challenge (Lucas et al., 2013) and protected cerebral tissue oxygenation (Anderson et al., 2019; Anderson et al., 2021). Mathematical modeling studies suggest that these BP oscillations could create a pump-like effect in the microvasculature extending perfusion of oxygenated blood further into tissues (Tsai and Intaglietta, 1993; Goldman and Popel, 2001; Hapuarachchi et al., 2010). In addition, clearance of interstitial fluid increased with induced vasomotion in the 0.02–0.12 Hz range in rabbit (Sakurai and Terui, 2006) and mouse models (van Veluw et al., 2020). These studies demonstrate that increased BPV in the LF range is beneficial, suggesting that treatments should enhance rather than reduce it.

Although speculative, our findings about relationships between LF-BPV and sociodemographic and biomedical variables are consistent with these mechanisms and their relationship to neurodegenerative disorders. In contrast to VVV-BP and ABPV, LF-BPV was inversely related to age, consistent with age-related increased risk of AD and other dementias. Similarly, in contrast to VVV-BP and ABPV, LF-BPV was greater in men compared to women, consistent with greater cognitive decline in women compared to men (Ferretti et al., 2018). Finally, in contrast to VVV-BP and ABPV, LF-BPV was negatively associated with the odds of having elevated CRP and with levels of IL-6, consistent with protection against AD (Zhang et al., 2022) and vascular dementia (Custodero et al., 2022).

Epidemiologic studies long ago established hypertension as an independent risk factor for coronary artery disease, stroke, and renal failure. A meta-analysis of 61 studies including almost 1,000,000 adults showed that across the age spectrum, the relationship between BP and heart disease mortality is consistent and continuous (Lewington et al., 2002). According to the prevailing view, the adverse effects of hypertension derive from elevated mean BP and variation around the mean merely represents “noise” to be disregarded, and the primary aim of treatment is to reduce mean BP. We now know that BP fluctuations contain information that may have important prognostic, therapeutic, and physiologic significance. Only further study of BPV in its various time scales will determine the degree to which this is the case.

## Data Availability

The datasets presented in this study can be found in online repositories. The names of the repository/repositories and accession number(s) can be found below: https://www.icpsr.umich.edu/web/ICPSR/series/203.
